# Influence of General and Local Anesthesia on Postoperative Pain after Impacted Third Molar Surgery

**DOI:** 10.3390/jcm10122674

**Published:** 2021-06-17

**Authors:** Jeong-Kui Ku, Jae-Young Kim, Mi-Kyoung Jun, Yeong Kon Jeong, Jong-Ki Huh

**Affiliations:** 1 Department of Oral and Maxillofacial Surgery, Gangnam Severance Hospital, Yonsei University College of Dentistry, Seoul 06273, Korea; KUJK@yuhs.ac (J.-K.K.); KJY810927@yuhs.ac (J.-Y.K.); 2 Sae-e Dental Clinic, 109-8, Songwon-ro, Jangan-gu, Suwon 16294, Korea; mijjomg@naver.com; 3 Department of Oral and Maxillofacial Surgery, Section of Dentistry, Armed Forces Capital Hospital, Armed Forces Medical Command, Seongnam 13634, Korea; ykjung5111@hanmail.net

**Keywords:** general anesthesia, impacted tooth, postoperative pain, third molar, tooth extraction

## Abstract

This study examined the effects of general anesthesia on the postoperative pain level after third molar extractions compared to local anesthesia. This retrospective study included patients who underwent four simultaneous third molar extractions under general or local anesthesia and had records of their postoperative pain levels (visual analog scale, VAS). The pain level was determined in the early (Postoperative day; POD < #3) and late (POD #3-7) periods. The operation time and recently modified difficulty index were analyzed to validate the homogenous condition of the extraction. Of the 227 male inpatients (aged 20.9 ± 1.3 years), 172 and 55 patients underwent third molar extractions under local and general anesthesia, respectively. The age and difficulty index were distributed equally, but the operation time was longer in general anesthesia than in local anesthesia (*p* < 0.001). The early and late periods featured similar pain outcomes. The operation time correlated with the total periods with a correlation coefficient of 0.271 (*p* < 0.001). In conclusion, the postoperative pain following whole third molar extraction was related to the operation time rather than the anesthetic methods.

## 1. Introduction

Of all dental procedures, third molar extraction causes the highest level of patient anxiety [1,2,3]. After a third molar extraction, the most frequent postoperative complication is pain, which is the primary reason for the anxiety [4,5]. The degree of anxiety shows a strong correlation with the postoperative pain level after the extraction [6]. Therefore, it is essential to reduce anxiety through intraoperative music or sedative medicine [4]. In general, third molar extraction is performed on one side with two extractions. On the other hand, unpleasant experiences related to the first extraction, such as fear, pain, and repeated surgical trauma, could provoke further anxiety and discomfort during the subsequent surgery [7].

General anesthesia is performed in patients who feel severe anxiety about dental treatment or suffer from the vomiting reflex or when surgery is required in a region adjacent to an anatomically dangerous structure. Furthermore, it was reported that dental surgery performed under general anesthesia minimizes anxiety and gives great satisfaction to the patients [8,9,10]. Therefore, general anesthesia has been recommended for the extraction of whole impacted third molars if the patient feels severe anxiety about dental treatment or wants to extract the third molars of both sides at once.

Nevertheless, general anesthesia should be considered the last treatment option because it carries a relatively high risk to the overall health, opportunity costs, and time spent preparing the anesthesia [11]. Regarding the cost effectiveness, the effects of general anesthesia should be discussed on an available basis. There is little evidence of postoperative discomfort of whole tooth extraction surgery under general anesthesia compared to local anesthesia. A comparison of the anesthetic effects on third molar extraction should consider the high variation of anatomic structures around the third molar, extraction difficulty, and the age and sex of the patients [12,13].

Recent studies have suggested the modified difficulty indices according to the spatial relationship, which was subcategorized based on the angle between the long axis of the third molar and the adjacent second molar [12,13]. In 2021, Qiao et al. reported a high risk of serious postoperative symptoms, such as pain, associated with the operation time independent of the impaction status of the mandibular third molar [14]. Regarding the effects of anesthetic methods, the postoperative pain associated with third molar extraction should be analyzed according to the difficulty index and the operation time.

The authors hypothesized that the anesthetic methods do not affect postoperative pain after extraction surgery. This study analyzed the effects of general anesthesia on the postoperative pain level after whole third molar extraction compared to local anesthesia.

## 2. Materials and Methods

The Institutional Review Board at the Armed Forces Capital Hospital approved this retrospective study (AFCH-20-IRB-034). The study was conducted according to the principles of the Declaration of Helsinki for research on humans. All the patients included in this study were adult inpatients who underwent four third molar extractions on both sides from January 2013 to November 2017 and had records on the postoperative pain levels of patients.

The inclusion criteria were as follows: (1) inpatients aged 19–24 years; (2) extraction of whole impacted third molars (both upper and lower third molars) at once under general or local anesthesia in the operating room; (3) informed consent from voluntary participants. The exclusion criteria were as follows: (1) uncontrolled systemic disease or dentofacial syndrome; (2) untreated dental caries or periodontitis; (3) related pathologic conditions, such as odontogenic cyst or pericoronitis; (4) heavy smoker (≥12 cigarettes per day).

### 2.1. Anesthesia and Surgery Process

Each patient was transported to the operating room without premedication with antibiotics and monitored with a non-invasive blood pressure monitor, a pulse oximeter, and a three-channel electrocardiograph. Local anesthesia involved infiltrating from five to eight 7.2 mL ampules of 2% lidocaine HCl (Huons, Seongnam, Korea) on the buccal vestibule and the palatal or lingual mucosa of the impacted third molars. General anesthesia was induced by an intravenous injection of remifentanil using a TCI system (Orchestra^®^ Base Primea; Fresenius Kabi, Bad Homburg vor der Höhe, Germany), 60 mg lidocaine, 160 mg propofol, and 50 mg rocuronium. The patient’s trachea was intubated with a nasal endotracheal tube (Portex Ivory, North Facing, Nasal, Profile Soft Seal Cuff, Polar Preformed Endotracheal Tube; Smiths Medical International Ltd., Hythe, UK) with an inner diameter of 7.0 mm. Oxygen and air were supplied at a fraction of inspired (FiO2) oxygen of 0.5 with sevoflurane. During anesthetic maintenance, the sevoflurane concentration was controlled so that the blood pressure and heart rate were held within 20% of the preoperative measurements; remifentanil was injected continuously for analgesia where necessary. Additional local anesthesia with 2–4 7.2 mL lidocaine ampules was also infiltrated on the buccal vestibule and the palatal or lingual mucosa of the impacted third molars. After surgery, sevoflurane and air were discontinued. In the immediate postoperative period, ketorolac was injected as a rescue analgesic. Pyridostigmine and glycopyrrolate were injected to reverse muscle relaxation when the patient could breathe spontaneously and respond to verbal commands.

Three expert oral and maxillofacial surgeons (extraction experience ≥ six years) performed the surgery. After exposing the mandibular third molar with or without an additional mesial or distal incision, an odontotomy was performed using a 2.0-mm round burr with a RemB straight microdrill (Stryker, Portage, MI, USA). After curettage of the remaining granulation tissue, a collagen plug (AteloPlug^®^, Hyundai Bioland, Cheongju, Korea) was packed to prevent food packing and bleeding. The patients were instructed to take oral antibiotics (625 mg amoxicillin, Ilsung Pharmaceutical, Seoul, Korea) and NSAIDs (500 mg dexibuprofen, Samil Pharmaceutical, Seoul, Korea) three times daily for five days and a daily mouth rinse with a chlorhexidine solution.

### 2.2. Postoperative Pain Analysis

The postoperative pain level was measured using a 10-cm visual analog scale (VAS, line from 0–10). The VAS from the day of surgery (postoperative day; POD #0) to the seventh day (POD #7 day) was investigated at 7 PM after dinner by a nurse. The maximum pain level was determined in the early (POD < #3) and late (POD #3–7) periods.

**Figure 1:**
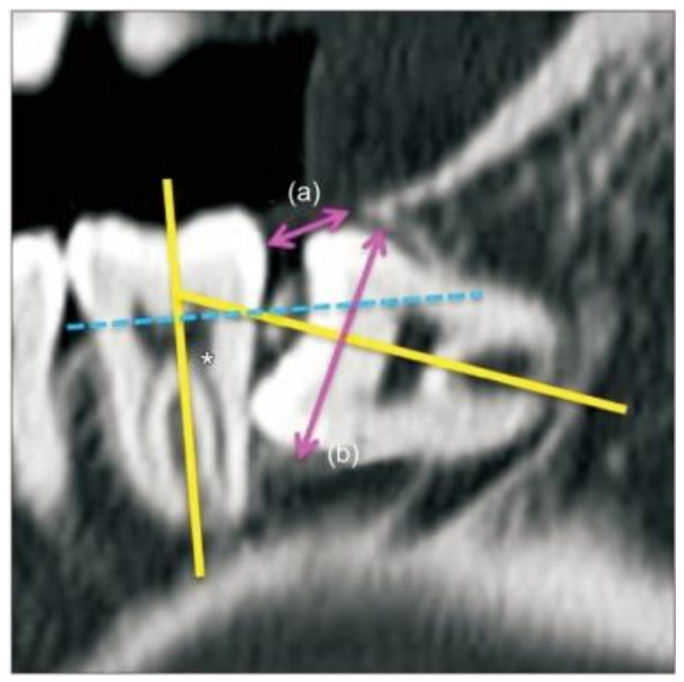
Measurement and classification of the impacted third molars in cone-beam computed tomography [12]. The spatial relationship was subcategorized based on the angle between the long axis of the third molar and the adjacent second molar as follows (yellow lines and an asterisk mark): (1) mesioangular (11° to 79°), (2) horizontal (80° to 100°), (3) vertical (−10° to 10°), (4) distoangular (−11° to −79°), or (5) reverse, where the crown of the third molar was more root-oriented than horizontal. The depth was classified based on the line connecting the cementoenamel junction of the adjacent second molar (dotted blue line) as follows: (1) more than half of the third molar crown was above the CEJ of the adjacent second molar; (2) less than half of the third molar crown was above the CEJ of the adjacent second molar; (3) more than half of the third molar crown positioned superior to the mid-level of the adjacent second molar root; (4) the third molar crown level inferior to that mentioned above. The ramus relationship/space available was subcategorized based on the ratio between the distance from the ascending ramus to the distal of the second molar (**a**) and the diameter of the impacted third molar (**b**) (pink arrows). An eruption space (**a**/**b**) larger than two-thirds of the distance was defined as (1), between one-third and two-thirds—as (2), and smaller than one-third—as (3).

The homogenous condition of the extraction surgery was validated by analyzing the operation time and recently modified difficulty index according to the anesthetic methods using an independent *t*-test. The operation time was defined as the time from the start of the incision to the last suture. Briefly, the difficulty scores for the mandibular third molars were defined considering the spatial relationship (1–5 points), depth (1–4 points), and ramus relationship (1–3 points) using cone-beam computed tomography (Figure 1). The difficulty index was defined as the sum of the difficulty scores: I (3–4 points), II (5–7 points), III (8–10 points), or IV (11–12 points) [12]. The higher index was used as the difficulty index of one patient compared to another.

According to the anesthetic methods, the difficulty indices were analyzed using Pearson chi-squared test. The age, operation time, and VAS were analyzed using an independent *t*-test. The relationship of the postoperative pain score to the difficulty index was analyzed by one-way ANOVA, to the operation time—using Pearson correlation coefficients. The data are presented as the means ± standard deviation, and the statistical analysis was performed using SPSS 25.0 for Windows (SPSS Inc., Chicago, IL, USA).

## 3. Results

Of the 227 inpatients, all the patients were male, with the mean age of 20.9 ± 1.3 years. One hundred seventy-two and 55 patients underwent third molar extraction under local and general anesthesia, respectively. The average age was similar between the anesthetic methods (20.9 ± 1.3 and 20.7 ± 1.0 years, respectively). Under general anesthesia, the operation time was longer (44.07 ± 19.09 min) than under local anesthesia (25.82 ± 13.31 min, *p* < 0.001), but the difficulty index was similarly distributed between the anesthetic methods (Table 1).

Between the local and general anesthetic methods, the postoperative pain score on the VAS was similar in the early (1.92 ± 1.60 and 1.98 ± 1.74, respectively) and late (0.54 ± 1.03 and 0.87 ± 1.40, respectively) periods (Table 2).

**Figure 2:**
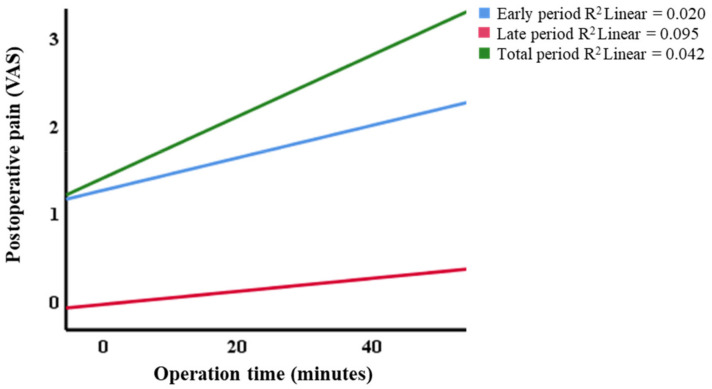
Postoperative pain level depending on the operation time. Postoperative pain score showed a significant correlation with the operation time in the early (R^2^ = 0.020, *p* = 0.035), late (R^2^ = 0.095, *p* = 0.000), and total (R^2^ = 0.042, *p* = 0.006) periods.

The difficulty index was unaffected by the postoperative pain VAS during the early period, late period, or when considering the total period (Table 3). Regarding the operation time, the postoperative pain level was significant in all the periods (*p* < 0.05). In particular, the operation time and the total period of pain correlated with a correlation coefficient of 0.271 (*p* < 0.001, Table 4, Figure 2).

## 4. Discussion

Many organizations have published guidelines regarding general anesthesia in dentistry [15,16]. General anesthesia is considered based on the patient’s preferences in addition to the particular indications, such as aggressive surgical procedures, the overall health of the patient, and uncontrolled behavior [17,18]. On the other hand, general anesthesia should be considered conservatively because of the risk to the patient’s overall health and cost [11]. To enhance satisfaction during a third molar extraction, general anesthesia has been generally recommended for patients with severe anxiety to prevent stress from intraoperative pain [4,6,8,9,10]. Nevertheless, the validation of general anesthesia has not been reported in clinical research with a comparison with local anesthesia. This study hypothesized that postoperative pain could be similar in local and general anesthesia, even though patients with general anesthesia did not feel anxiety and stress during the procedure.

Although third molar extraction is the most common surgery for oral surgeons, there has been a lack of research on the extraction due mainly to the highly divergent morphology of the third molar and surrounding structures. Furthermore, age, smoke, and sex also affect the difficulty and prognosis of the extraction [19,20,21]. Regarding the third molar research, a Korean military hospital has several advantages: most patients are male; 92.0% of all enlisted Korean soldiers are under 22 years of age; medical treatment is free of charge for all active-duty patients; there is a high proportion of third molar patients [22]. Therefore, this retrospective study could compare the age, sex, and difficulty indices between anesthetic methods. Considering the similar distribution with age, sex, and difficulty indices, the postoperative pain score was unaffected by the anesthetic methods during the early and late periods (Table 2).

Although the pain score did not show a difference between the anesthetic methods, this study analyzed the relationship between the difficulty index and operation time regarding other factors contributing to postoperative pain. The difficulty index did not have any significant effect on postoperative pain. On the other hand, pain correlated with the operation time in all the periods (*p* < 0.05). The postoperative pain was most related to the sum of pain during the entire follow-up period with a correlation coefficient of 0.271 (*p* < 0.001). This result was in accordance with the previous research showing that prolonged operation time could increase postoperative complications [23,24,25,26].

Various factors can affect the operation time, such as tooth anatomy, the patient’s position, various surgical methods, and the surgeon’s condition and experience. Although validation of the difficulty index has been reported in a recent study [12], the operation time differed according to the anesthetic method regardless of an evenly distributed difficulty index of the third molar. Furthermore, prolonged operation time could increase postoperative complications [23,24,25]. Under general anesthesia, the operation time could be longer because the patients could not cooperate with mouth opening and positioning, even though additional local anesthesia was unnecessary [27]. In conclusion, postoperative pain under general anesthesia could not be reduced after four third molar extractions compared to surgery under local anesthesia. To decrease postoperative pain, the surgeon should consider reducing the operation time.

This retrospective study had several limitations, such as a heterogeneous extraction process, comparison with outpatients, and absence of subjective satisfaction, including the postoperative pain, masticatory function. In this study, the straight air drill used in the operation room had a higher torque than the low-speed handpiece and a different access entrance to the surgical field from the high-speed handpiece at the dental chair. Further prospective research will be needed to reveal the relationship between the operation time and postoperative pain and the difference between the extraction in an operating room and the outpatient chairside with controlled incision design and subjective satisfaction.

## 5. Conclusions

General anesthesia could not reduce the postoperative pain after the extraction surgery of an impacted third molar. On the other hand, prolonged operation time could be related to the degree of postoperative pain.

## Figures and Tables

**Table 1:** Clinical information of the extraction patients according to the anesthetic method.

	Total (*n* = 227)	Anesthetic Method		
		Local Anesthesia (*n* = 172)	General Anesthesia (*n* = 55)	*p*
Age (years)	20.9 ± 1.3	20.9 ± 1.3	20.7 ± 1.0	0.447
Operation time (minute)	30.24 ± 16.81	25.82 ± 13.31	44.07 ± 19.09	<0.001 *
Difficulty index (*n*, %)				
I	28 (12.3%)	22 (12.8%)	6 (10.9%)	0.901
II	157 (69.2%)	119 (69.2%)	38 (69.1%)	
III	42 (18.5%)	31 (18.0%)	11 (20.0%)	

* *p*-value < 0.05.

**Table 2:** Postoperative pain level depending on the type of anesthesia.

	Postoperative Pain (Visual Analog Scale)		
	Local Anesthesia (*n* = 172)	General Anesthesia (*n* = 55)	*p*
Early period (POD < #3)	1.92 ± 1.60	1.98 ± 1.74	0.823
Late period (POD #3–7)	0.54 ± 1.03	0.87 ± 1.40	0.065

POD, postoperative day.

**Table 3:** Postoperative pain level depending on the difficulty index.

	Difficulty Index			
	I	II	III	*p*
Postoperative pain (VAS)				
Early period (POD < #3)	1.77 ± 1.27	1.92 ± 1.60	2.14 ± 1.95	0.584
Late period (POD #3–7)	0.81 ± 1.39	0.61 ± 1.13	0.59 ± 1.02	0.694
Total period	2.67 ± 3.01	2.45 ± 2.78	2.44 ± 2.33	0.942

VAS, visual analog scale; POD, postoperative day.

**Table 4:** Correlation coefficient analysis between the operation time and postoperative pain level.

	Operation Time	
	Co	*p*
Postoperative pain level (VAS)		
Early period (POD < #3)	0.140	0.030 *
Late period (POD #3–7)	0.201	0.004 *
Total period (POD #0–7)	0.271	< 0.001 *

VAS, visual analog scale; POD, postoperative day; Co, correlation coefficient; * *p* < 0.05.

## Data Availability

Not available.
